# Supportive care of neurodegenerative patients

**DOI:** 10.3389/fonc.2023.1029938

**Published:** 2023-02-14

**Authors:** A. E. Armitage, E. Fonkem

**Affiliations:** ^1^ Department of Supportive Palliative Care, Baylor Scott and White Health, Temple, TX, United States; ^2^ Department of Neurosurgery, Baylor Scott and White Health, Temple, TX, United States

**Keywords:** glioblastoma, supportive care, Parkinson’s disease, neurodegenerative disease, palliative care

## Abstract

Neurodegenerative illnesses are notorious for paucity of treatments and relentless clinical progression. Illness may follow a relatively acute presentation, as is seen with primary brain tumors such as glioblastoma or have a more insidious onset with a slower yet unyielding course, such as that seen in Parkinson’s disease. Though disparate in presentation, these neurodegenerative illnesses are universally terminal, and both the patients and their families benefit from the intervention of supportive care in conjunction with primary disease management. Supportive palliative care has been shown to improve quality of life, enhance patient outcomes, and often extend patient life—but such care needs to be tailored. This clinical commentary examines the role of supportive palliative care in the management of neurologic patients, comparing and contrasting glioblastoma patients with idiopathic Parkinson’s disease patients. Both patient populations are high utilizers of healthcare resources, require active management of multiple symptoms, and have high caregiver burden which underscores the need for supportive services in conjunction with disease management provided by the primary care team. Review of prognostication, patient and family communication, trust and relationship building, and complementary medicinal approaches are explored for these two diseases which broadly represent two differing poles of incurable neurological illness.

## The two poles of neurodegenerative disease

The needs of neuro-oncology patients with serious illness are unique and differ from other terminal illnesses and particularly from other neurodegenerative disorders. When considering neuro-oncologic illnesses such as glioblastoma (WHO grade IV), the trajectory of the disease is short and aggressive, characterized by rapid functional and cognitive impairment (1, 2). The diagnosis is given when the disease is more advanced. An early palliative care referral is recommended and set as standard of care by the American Society of Clinical Oncology to improve quality of life, ensure a dignified death, and ease caregiver burden and grief (3, 4). In contrast, other neurodegenerative disorders such as Parkinson’s disease may smolder for years before active symptoms present, and the progression is slow and proceeds over an extended period. These differences in disease progression and prognosis underscore the vastly different approaches needed in palliation of terminal neurological disorders. This article is a professional commentary on neurodegenerative disease in a palliative setting based on the authors’ clinical observations and personal experience from three established subspecialty departments (neuro-oncology, movement disorders, and palliative care) in a Level 1 medical center in central Texas USA and supported by the evidence-base. We will look at glioblastoma and Parkinson’s disease patients as archetypal of rapid versus protracted neurodegenerative disorders, comparing and contrasting their unique end of life needs. The diagnosis, progression and prognostication for these two diseases will be compared and contrasted, as well as an in-depth look at caregiver burden. Discussion on individual symptom management is beyond the scope of this paper.

## The diagnosis, progression, and prognostications of the two diseases

The diagnosis of glioblastoma, after symptom presentation, is a grade 4 diagnosis with a prognosis of 15-18 months assuming aggressive medical intervention (5), Hemminger et al. (2) note a mean survival of 12.6 months. For the glioblastoma patient, palliative care should be engaged early (3) ideally on first diagnosis, regardless of presenting symptomology due to the aggressive nature of the disease and overall poor prognosis. Patients move from independent to fully dependent over months, requiring rapid shifts in how the patient views themselves and how they function day to day. Due to the speed of disease progression, relationship building and trust development between clinician, patient and patient’s family needs to be accomplished quickly. The focus is therefore on clear and compassionate communication, with frequent supportive medical interactions resulting in rapid confidence building.

At time of presentation in Parkinson’s disease there is already a 60% dopaminergic neuronal loss in the substantia nigra (6), but it may be a decade before disability becomes significantly life-limiting. Death may be anticipated 15 – 26 years from first prognosis, with disability occurring in a non-linear fashion (7, 8). Parkinson’s disease is distinctive of other neurological disorders in the wide presentation of symptomology and variability in prognosis at time of diagnosis (9). Palliation of symptoms early in Parkinson’s disease may be provided by the primary neurological treatment team and specialist palliative care is appropriately called in many years after first diagnosis for more complex symptom management.

Communication in both a disease that has a rapid decline and one that has a prolonged decline can be aided by structured information sharing. There are several communication models and frameworks that can aid the provider in navigating these complex skills and practices (10, 11). The Ask-Tell-Ask method is one such method and involves first asking the patient to share their understanding of their disease (12, 13). This sharing allows the provider to assess what level of communication is needed in patient education and helps prevent repeating information that the patient already knows. The “Tell” then allows the provider, with permission, to share further information on the disease and prognosis personally tailored to the patient based on their initial disease description. The presence of uncertainty in prognostication and the limitation of prognostic tools should also be communicated. The last “Ask” allows the palliative provider to reassess patient understanding, correct misconceptions and fill in gaps in knowledge (14).

Glioblastoma patients benefit from decision making addressed up front, while the patient still has the cognitive abilities to understand and make their own decisions. Advanced directives should be completed soon after diagnosis, and it is preferable for the family to be brought in to hear the patient’s preferences firsthand. The loss of cognitive abilities in Parkinson’s disease is slower and can be anticipated, allowing the patient’s wishes to be documented with ample time while the patient is still competent to make their own decisions.

Although life expectancy for glioblastoma patients has increased with the advent of new therapies, the outlook for most patients is still dire. Glioblastoma is well understood; however, many patients have poor awareness of disease trajectory and short life-expectancy (15). Good prognostic awareness is associated with a more favorable quality of life for the patient and reduced psychosocial stress for the caregiver (16). Prognostication is more complex in Parkinson’s disease due to the longer disease trajectory, variable stages of disease at diagnosis, non-linear manifestation of symptoms and other comorbidities which have more opportunity to impact disease progression and overall patient disability. Unlike glioblastoma patients, Parkinson’s patients can improve with exercise, which is known to modify disease progression (17). Optimized sleep, nutrition, stress control can help manage symptom manifestation, and patient engagement in disease modifying life-style changes can further challenge prognostication for the Parkinson patient.

Symptoms that affect social participation, reduced mobility, reduce ability to perform activities of daily living, and increase depression in both patient populations have a profound effect on patient quality of life. Discussion on symptom management for both the glioblastoma patient and the Parkinson’s patient is outside of the scope of this discussion, and there are some excellent articles to which we would refer the reader (6, 7, 18–20). In addition to disease treatment and symptom management, we would offer that patient and caregiver support in these areas are foundational to adaptation to disease. Having a clear understanding of the disease processes, expected progression and how to adapt to the changing landscape normalizes change and can significantly enhance acceptance and provide emotional peace and resiliency on both the part of the patient and the care giver. A reasonably recent framework for the support and palliation of patients with high grade gliomas was proposed by Philip et al. (21) which is an interesting guide for provider teams and worth exploring. We hope more research will be done in this area to support their findings.

## Patient and family education

A patient’s understanding of their disease and treatment options is foundational to their ability to make educated and meaningful medical decisions. Posing such conversations in the “Hope and Worries” framework preserves the crucial element of hope that impacts a patient’s ability to face the future and share their worries. This allows for a greater understanding of the realities of the disease and leaves the patient with a realistic perspective in which to plan, so that the element of surprise is limited. The literature suggests that patients prefer prognostic information communicated in a manner that preserves hope (1). Patients vary in their wishes to know their prognosis and in the level of detail they want. Ariadne Labs’ Serious Illness Conversations Program (https://www.ariadnelabs.org/serious-illness-care/) may serve as foundational when beginning to understand and care for the patient on their own terms. They have developed clinician conversation guides, patient and caregiver workbooks and resources to guide these difficult conversations. Conversations can start simply with asking the patient to share what they understand about their diagnosis, what their treating provider shared about prognosis and how much information they would be comfortable receiving (big picture, detailed information or only the good news.) It helps to explore the benefits of having such conversations when the patient and provider have the time and space to do this in a thoughtful and calm manner, emphasizing that beginning such conversations during a time of crisis is incredibly challenging.

Building relationships with Parkinson’s patients spans months to years. The Parkinson’s communities nationally are well established with patient resources and support for care givers. There is active outreach by national and international organizations such as the Parkinson’s Foundation (https://www.parkinson.org/), Michael J Fox foundation (https://www.michaeljfox.org/) The Davis Phinney Foundation (https://davisphinneyfoundation.org/, Parkinson’s UK (https://www.parkinsons.org.uk/information-and-support), Parkinson’s Europe (https://www.parkinsonseurope.org/) where patients can educate and empower themselves and come prepared to their medical appointments.

In a similar fashion there are online resources for brain cancer patients. The Glioblastoma Foundation (https://glioblastomafoundation.org/), and the Glioblastoma Support Network (https://glioblastomasupport.org/) offer glioblastoma patients and families patient-focused education and support in the USA and worldwide. The International Brain Tumor Alliance (https://theibta.org/brain-tumour-support-advocacy-and-information-organisations/) has a worthwhile listing of support groups and organizations internationally. Overall, there are fewer universal sources of patient-centered education for glioblastoma patients, which results in a patient relying more heavily on their clinicians, nurses, and other members of their interdisciplinary medical team for disease education. To support the patient fully, follow-up appointments are closer together than for the Parkinson patient. The high healthcare utilization underscores the need for supportive palliative care to be involved right from the time of diagnosis to walk the disease path with the patient, provide education and symptom control. This sharing of load reduces the burden on the rest of the healthcare team.

## Caregiver burden

### Burden as a function of disease progression

Caregivers in both Parkinson’s disease and glioblastoma can suffer a lot of stress and the burden can be high. Regardless of the rapidity of the disease process, there are changes in relationships and roles due to disability and increased dependency on family and caretakers (22). In glioblastoma the stressors include hasty adaptation to rapid changes in patient capabilities, and less time to be educated and connect with others who have a similar disease to understand the benchmarks, norms, and prognosis of the glioblastoma patient. With the slow evolution of Parkinson’s disease and a robust national social and educational infrastructure, there is greater time for adaptation, education and understanding. Although the long duration of disease wears heavily on caregiving, this eases the pressure on the clinician to provide all the education, and it allows the caregiver time to adapt to change. The slow progression of Parkinson’s disease also allows space for discussion around the disease, treatments, and current and future available research.

### Burden due to disease complexity

There may be complex needs at any stage during neurological illness. Parkinson’s patients may present with very variable symptomology and have a wide variety of care requirements (9) which results in the Parkinson patient and caregiver leaning on the provider to normalize expectations and provide a more tailored vision of what to expect in the future. Complexity of care may depend on the ability and skills of the caregiver (22). Motor symptoms define Parkinson’s disease: tremor, slowness, rigidity and, later in the disease, falls. In a similar fashion, glioblastoma patients are hampered by reduced mobility as their disease progresses, depending on their tumor location. Caregivers contend with reduced mobility which affects not only the patient’s quality of life, but also increases the caregiver demand in time and attention, as well as physical strain. Non-motor symptoms of the disease can be as much, if not more burdensome to the caregiver (23). Symptoms such as rem sleep behavior disorder, constipation, micrographia, apathy, anxiety and depression are seen early on in disease presentation often before the diagnosis is fully fleshed out. As non-motor symptoms in Parkinson’s disease often present well before motor manifestations of the disease, it adds complexity to the diagnosis, understanding and care for the patient (9). Being a caregiver in any relationship can be difficult and may come with negative health outcomes (24). Parkinson’s caregivers have been found to have higher rates of depression, increased susceptibility to illness and poorer quality of life even when compared to caregivers in other diseases (25). This may be in part related to the duration of the disease and the slow, but relentless patient decline over years which affords the caregiver little respite. The interdisciplinary team in palliative care practice can attend to caregivers in addition to the patient’s needs. Social work, chaplain services, child life specialists and psychology are core in this integrative support, be it an intense and rapid decline of a glioblastoma patient or the relentless decline of the Parkinson’s patient over years wearing the caregiver down.

### Assessing disease burden

There are numerous validated scales that can help measure and quantify caregiver burden. There are three common scalers: The Zarit Caregiver Burden Inventory (26), The Caregiver Burden Inventory (27) and The Caregiver Strain Index (28). The scales are designed to assess the level of burden that the caregiver experiences for the patient, caregiver, and provider to have a better understanding of how quality of life can be improved.

### Signs of caregiver burden

Signs of caregiver burden include: Denial of the severity of the disease or management; Anger at the disease or towards the patient, or difficulty managing emotions; Social withdrawal from hobbies, social support, or activities; Anxiety about the future, the unexpected or even day-to-day routines; Depression impacting perceptions as well as the ability to cope with stress; Fatigue, lacking energy to get through each day; Poor sleep which may be due to poor sleep hygiene or interruptions in sleep to provide care; Difficulty concentrating, completing tasks, or staying focused; Health problems, possibly because of deferring their own care for the care of the patient.

### Interventions for the care giver

There is an increasing awareness of the value of specific caregiver training in the management of both cancer and neurological patients. The support groups discussed above have sections devoted to caregiver education and support, and research is emerging on the benefits of informal training by the patient’s professional team. Empowerment of both the patient and the caregiver are key which includes the “caregivers’ confidence that they can help alleviate their loved ones’ symptoms” (29–31). Interventions for the caregiver may include such things as training caregivers in daily care skills, medication management, mechanics of movement to reduce patient falls and improve mobility. Caregivers can experience strain in multiple dimensions including emotionally, socioeconomically and financially, and caregiver strain is also known to be influenced by their attitude and sense of empowerment (32). Interventions should include empathic listening and asking open ended questions by all members of the palliative interdisciplinary team to better understand caregiver pressure points, as not all patients will experience all symptoms and resources should match their needs (33, 34). Caregivers can be encouraged to use mind-body techniques for stress reduction and mood control (35). A well-rounded palliative program may offer caregiver support groups to normalize some of these feelings and experiences. Caregivers can be encouraged to take time for self-care and utilize respite care services, as it is the intensity (number of hours) of caregiving that correlates to caregiver strain (33). The provider should also consider optimizing the patient’s medication regimen to appropriately control symptoms which makes caretaking easier and thus reduces burden.

### Grief

Processing grief in both the Parkinson patient and the glioblastoma patient is complex and multifactorial. Much of this occurs with and through the patient’s family, community and foundational support systems, for example the patient’s primary care providers or spiritual leaders. This burden of processing for both the patient and the family, which is deep and often complex, can be further supported by interdisciplinary palliative services including psychology, child life specialists, social work and chaplains.

Due to the long disease trajectory of Parkinson’s disease, anticipatory grief is possible and common (36). Patients and caregivers have time to work through the mourning process for lost functionality and separation from loved ones through impending death. Palliative psychologists may lead a patient and their caregivers through these feelings and bring peace to the space that the patient occupies. Seyama and Kanda (37) elegantly describe the challenge that families face when living with ““ conflicting emotions that never go away” [which] are present from the time of diagnosis until death and that the family must live while reconciling the polar emotional states of hope and pain”. In glioblastoma patients, due to the rapidity of the disease process there is less time to come to terms with the diagnosis, change, fear, loss and death. Just the concept that the disease is rapid and relentless can be overwhelming, with the knowledge that there is little time to achieve life goals. The palliative health psychologist’s role focuses more on acceptance of diagnosis and preparation for end-of-life, which is done in months, not years.

Good prognostic awareness from time of diagnosis can help with caregiver grief (16) Child life specialists become especially critical in families with children involved with the patient. The rapid decline of a loved one can be frightening and poorly understood. Children notice change, disease and decline even when not spoken about if they do not have context, they may create their own interpretation of what is happening to the patient or assign blame inappropriately. Child life specialists help children understand change and come to terms with the impending loss of a loved one in a non-threatening manner. Child life can also work on legacy building with the patient and the child while the patient is still alive, leaving more than just memories to ease grief and memorialize the patient.

## Conclusion

Supportive palliative care is adjunct and integral to quality care of a terminally ill patient. Palliative care specialists function as an added layer of support for terminally ill patients. The patient is not required to change any treatment for their disease or treatment provider. While not interfering with active disease treatments, symptoms and side effects from the disease process and its treatment can be managed proactively. Engagement early in the disease process provides optimal patient care.

Engaging supportive palliative care is the engagement of a team. The medical provider may lead the patient and family care, but the true value of the palliative team is the sum of the individual members of the associated interdisciplinary team. A team consists of physicians, advanced practice providers, nurses, social worker, psychologist, chaplain, and child life specialist.

There are many similarities between the palliative management of a neurooncological patient, such as glioblastoma and a neurodegenerative patient such as Parkinson’s disease. The differences, although nuanced, are important to successful patient management.

## Author contributions

All authors listed have made a substantial, direct, and intellectual contribution to the work and approved it for publication.
